# Non-linear dose-response relationship between the visceral adiposity index and diabetes in adults with normoglycemia: a cohort study

**DOI:** 10.3389/fendo.2024.1441878

**Published:** 2024-12-04

**Authors:** Xiaomin Liang, Zemao Xing, Ying Li, Shuiqing Gui, Haofei Hu

**Affiliations:** ^1^ Department of Critical Care Medicine, Shenzhen Second People’s Hospital, Shenzhen, Guangdong, China; ^2^ Department of Nephrology, Shenzhen Second People’s Hospital, Shenzhen, Guangdong, China

**Keywords:** diabetes, visceral adiposity index, non-linear, normoglycemia, cohort study

## Abstract

**Objective:**

Previous studies have identified a positive link between the visceral adiposity index (VAI) and diabetes in specific populations. Our investigation focused on examining this association in normoglycemic adults in Japan.

**Methods:**

A cohort study of NAGALA (NAfld in the Gifu Area Longitudinal Analysis) was undertaken from 2004 to 2015 in Japan. The link between VAI and diabetes was evaluated using multivariate Cox proportional hazards regression and restricted cubic spline (RCS) regression models. Receiver operating characteristic (ROC) curve analysis was performed to assess the predictive value of the VAI for incident diabetes.

**Results:**

Our study included 15,452 participants, with 8,418 men (54.48%) and 7,034 women (45.52%). The average age was 43.71 ± 8.90, and 373 participants (2.41%) developed diabetes. VAI was positively related to diabetes (HR=1.13, 95% CI 1.08-1.18). The inflection point of the non-linear relationship was observed at a VAI value of 4.67. For the VAI values up to 4.67, one unit increase in the VAI related to a 24% increase in new-onset diabetes (HR=1.24, 95% CI 1.12-1.37, p<0.0001). Subgroup analysis detected a more robust relationship in women (HR=1.40, 95% CI 1.14-1.70, p=0.0010). ROC analysis indicated that VAI, with an AUC of 0.7479 (95% CI: 0.7237-0.7720), had good predictive power.

**Conclusion:**

Our cohort study validated the positive and non-linear relationship between the VAI and diabetes in normoglycemic adults in Japan. The relevance was more marked in women than in men. For those with a VAI below 4.67, a further reduction in the VAI could potentially lead to a significant decrease in diabetes risk.

## 1 Introduction

Globally, diabetes stands out as having extensive health effects caused by its widespread occurrence and high incidence. This condition increases the risk of physical impairments, cardiovascular ailments, and death rates (1–3). The International Diabetes Federation’s most recent survey report revealed that in 2021, the global adult population (ages 21-79) with diabetes was 536.6 million (4). This figure is expected to increase by 45.92% to 783 million by 2045 (4). Furthermore, the economic burden of diabetes is substantial and escalating, placing immense strain on both healthcare infrastructures and families (4).

Excessive or abnormal accumulation of fat, defined as obesity and overweight, poses a health risk to individuals (5). The global rise in diabetes is thought to be significantly influenced by an increase in obesity (6). Nevertheless, body mass index (BMI) alone may not adequately represent the risk of diabetes, as it does not account for the excess deposition of ectopic fat and visceral adipose tissue (7, 8). Methods utilizing waist circumference (WC) have been established to evaluate visceral fat levels (9). However, the limitation is that visceral fat cannot be distinguished from subcutaneous fat when solely using WC (10). Numerous studies indicate that visceral fat serves as a marker for atherosclerotic burden in individuals with metabolic issues, while subcutaneous fat appears to offer some protection (11, 12). It has been demonstrated in some research that visceral fat generates a higher quantity of free fatty acids, thereby increasing the likelihood of diabetes and insulin resistance (13, 14).

The visceral adiposity index (VAI) is a novel sex-specific index that is derived from measurements of triglycerides (TGs), high-density lipoprotein cholesterol (HDL-c), BMI, and WC, providing an indirect assessment of visceral fat function (15). Studies have suggested that the sensitivity of insulin and function of visceral fat are measured by VAI, with a higher score being intimately linked to cardiometabolic risk (15). Previous research has established that VAI is a reliable correlation indicator of non-alcoholic fatty liver disease (16), chronic kidney disease (17), cardiovascular disease (18), and metabolic syndrome (19). Some investigations into VAI in the context of diabetes have identified a positive correlation (20–23). However, these findings have not been validated in Japanese adults. Consequently, our investigation focused on the link between VAI and diabetes in normoglycemic adults in Japan.

## 2 Methods

### 2.1 Study data and population

The DATADRYAD database (http://www.Datadryad.org/) served as the primary source of our data. We accessed the information through the Dryad data package (https://doi.org/10.5061/dryad.8q0p192), which is accessible to all researchers at no cost. The website provides raw data that researchers can use for secondary analysis without copyright infringement.

**Figure 1:**
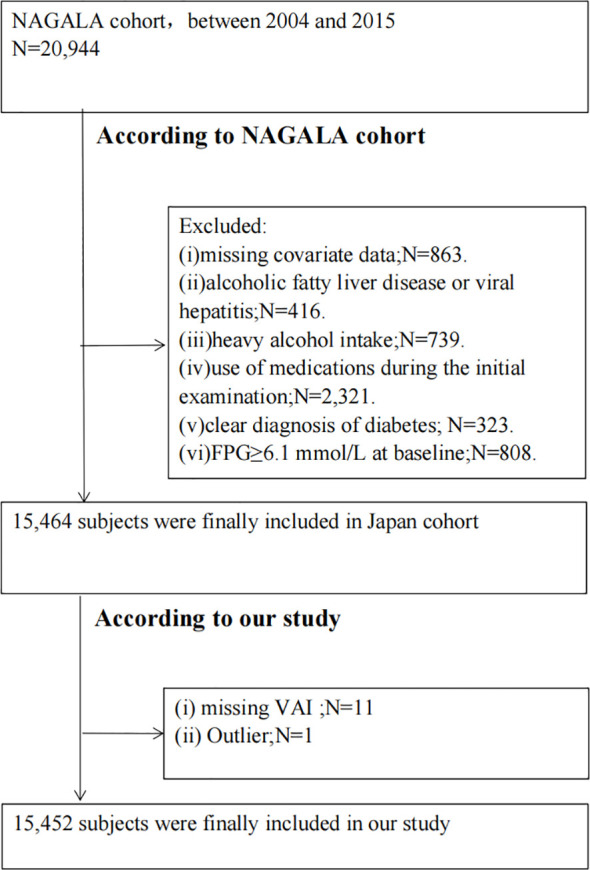
Flow chart of the study population.

Our study involved a secondary examination of the NAGALA (NAfld in the Gifu Area, Longitudinal Analysis) database (24). The cohort study took place at the Medical Health Checkup Center of Murakami Memorial Hospital in Gifu, Japan, from 2004 to 2015 (24). In summary, the original study recruited 20,944 individuals who had undergone at least two medical examinations between 2004 and 2015 (24). The original study excluded participants based on several criteria: (i) missing covariate data; (ii) alcoholic fatty liver disease or viral hepatitis; (iii) heavy alcohol intake (over 40 g/day for women, 60 g/day for men); (iv) use of medications at the initial examination; (v) clear diagnosis of diabetes; or (vi) fasting plasma glucose (FPG) at or above 6.1 mmol/L (24). Our study additionally excluded those with missing VAI data and outliers. Ultimately, 15,452 subjects were enrolled in our study. The ethics committee of Murakami Memorial Hospital granted approval for the investigation (24). Every participant supplied signed written consent permitting the use of their data (24). A flowchart of this process is depicted in **Figure 1**.

### 2.2 Data collection and measurements

All participants’ health backgrounds and habitual influences were collected using a standardized questionnaire that they completed themselves (24). Those who routinely participated in some form of exercise at least once per week were classified as regular exercisers (24). Skilled sonographers diagnosed fatty livers according to abdominal ultrasound findings (24). Baseline data provided us with relevant data on BMI, regular exercise, sex, diastolic blood pressure (DBP), alcohol status, WC, age, smoking status, systolic blood pressure (SBP), and the presence of a fatty liver. Baseline laboratory data included hemoglobin A1c (HbA1c), triglycerides (TGs), gamma-glutamyl transferase (GGT), total cholesterol (TC), high-density lipoprotein cholesterol (HDL-c), aspartate aminotransferase (AST), fasting plasma glucose (FPG), and alanine aminotransferase (ALT).

### 2.3 VAI and diabetes definitions

VAI is determined using separate formulas for men and women (15).

For men, the calculation is VAI=[WC/(39.68+1.88×BMI)]×(TG/1.03)×(1.31/HDL−c) (15).

For women, it is VAI=[WC/(36.58+1.89×BMI)]×(TG/0.81)×(1.52/HDL−c) . Here, BMI is measured in kg/m^2^, WC in cm, and HDL and TG in mmol/L (15).

The onset of diabetes is identified by HbA1c at or above 6.5%, FPG at or over 7 mmol/L, or through self-report (25).

### 2.4 Statistical analysis

Continuous variables are depicted as either median (interquartile ranges) or as a mean (standard deviation). In contrast, categorical data are shown as quantities (proportions). To compare differences in continuous data across VAI tertiles, we employed a one-way analysis of variance (ANOVA), while chi-squared tests were applied to categorical variables.

Covariates that exhibited a variance inflation factor (VIF) exceeding 5 were identified as collinear. To address the dose-response connection between the VAI and diabetes, we employed restricted cubic splines (RCS). The relationship between the VAI and diabetes was estimated using Cox regression models. The findings are given as hazard ratios (HRs) followed by 95% confidence intervals (CIs). Crude regression estimates and those adjusted for covariates are provided. The selection of confounders was influenced by their correlation with the outcomes or whether they could shift the effect estimates by over 10%. After taking into account their clinical relevance, we made adjustments for the following covariates: AST, age, GGT, SBP, ALT, sex, TC, regular exercise fatty liver, alcohol consumption, and smoking status.

We employed a two-piecewise Cox regression model to investigate the threshold impact of the VAI on diabetes (26). The inflection for the VAI was identified using a recursive method. Ultimately, the model that best explained the link between the VAI and diabetes was chosen, building on a log-likelihood ratio test.

Considering the strong effect of exercise, hypertension, and alcohol on diabetes, we conducted sensitivity analyses in participants without regular exercise, hypertension, and alcohol consumption to ensure that our findings were robust. We investigated the possibility of unaccounted confounding factors for the link between the VAI and diabetes by determining E-values (27). These E-values measure the strength of an unseen confounder that could potentially nullify the noted VAI-diabetes connection.

Receiver operating characteristic (ROC) curve analysis was used to determine the predictive value of the VAI, BMI, and WC for incident diabetes. The two-sided alpha level was set at 0.05. EmpowerStats and R software were utilized for all statistical calculations.

## 3 Results

### 3.1 Baseline characteristics

The initial attributes of individuals participating in our research are outlined in **Table 1**. The study comprised 15,452 subjects, of whom 54.48% (8418) were men and 45.52% (7034) women. The participants’ mean age was 43.71 ± 8.90, and diabetes was reported in 2.41% (373) of them. The VAI values ranged between 0.03 and 21.41, with a median of 0.78 (0.39,1.58). We categorized the VAI into three tertiles: T1 (0.03-0.49), T2 (0.49-1.22), and T3 (1.22-21.41). As we moved from T1 to T3, there was a noticeable increase in the values for SBP, HbA1c, WC, ALT, BMI, AST, age, TC, GGT, TG, FPG, DBP, and in the proportion of men, current smokers, individuals with a fatty liver, and alcohol consumers. Conversely, the proportion of women, regular exercisers, and HDL-c levels showed a decreasing trend. The incidence of diabetes escalated across tertiles, with rates of 0.56% in T1, 1.73% in T2, and 4.95% in T3 (**Table 1**).

**Table 1:** Baseline characteristics of the participants.

VAI tertile	T1(0.03-0.49)	T2(0.49-1.22)	T3(1.22-21.41)	P-value
Number of participants	5151	5150	5151	
Age, years	41.62 ± 8.43	44.42 ± 9.11	45.09 ± 8.76	<0.001
WC, cm	70.00 ± 6.87	76.33 ± 7.60	83.08 ± 7.63	<0.001
GGT, U/L	12.00 (10.00-15.00)	15.00 (12.00-21.00)	21.00 (16.00-32.00)	<0.001
AST, U/L	16.00 (13.00-19.00)	17.00 (14.00-21.00)	19.00 (15.00-23.00)	<0.001
ALT, U/L	13.00 (11.00-17.00)	16.00 (13.00-22.00)	22.00 (17.00-31.00)	<0.001
BMI, kg/m^2^	20.32 ± 2.36	22.04 ± 2.82	23.99 ± 3.01	<0.001
FPG, mmol/L	4.96 ± 0.39	5.18 ± 0.39	5.35 ± 0.37	<0.001
TG, mmol/L	0.44 ± 0.15	0.76 ± 0.23	1.53 ± 0.75	<0.001
TC, mmol/L	4.92 ± 0.81	5.09 ± 0.85	5.37 ± 0.87	<0.001
HDL-c, mmol/L	1.79 ± 0.37	1.46 ± 0.28	1.13 ± 0.23	<0.001
HbA1c, %	5.14 ± 0.30	5.17 ± 0.32	5.20 ± 0.34	<0.001
SBP, mmHg	107.86 ± 13.13	115.07 ± 14.31	120.55 ± 14.64	<0.001
DBP, mmHg	66.66 ± 9.17	71.85 ± 9.94	76.22 ± 10.12	<0.001
Sex				<0.001
Male	670 (13.01%)	3025 (58.74%)	4723 (91.69%)	
Female	4481 (86.99%)	2125 (41.26%)	428 (8.31%)	
Fatty liver				<0.001
No	5039 (97.83%)	4548 (88.31%)	3128 (60.73%)	
Yes	112 (2.17%)	602 (11.69%)	2023 (39.27%)	
Regular exerciser				<0.001
No	4234 (82.20%)	4179 (81.15%)	4334 (84.14%)	
Yes	917 (17.80%)	971 (18.85%)	817 (15.86%)	
Alcohol consumption				<0.001
None	4518 (87.71%)	3820 (74.17%)	3463 (67.23%)	
Light	357 (6.93%)	666 (12.93%)	731 (14.19%)	
Moderate	235 (4.56%)	471 (9.15%)	651 (12.64%)	
Heavy	41 (0.80%)	193 (3.75%)	306 (5.94%)	
Smoking status				<0.001
Never	4263 (82.76%)	2947 (57.22%)	1817 (35.27%)	
Past	498 (9.67%)	1074 (20.85%)	1377 (26.73%)	
Current	390 (7.57%)	1129 (21.92%)	1957 (37.99%)	
Diabetes incident				<0.001
No	5122 (99.44%)	5061 (98.27%)	4896 (95.05%)	
Yes	29 (0.56%)	89 (1.73%)	255 (4.95%)	

Categorical variables were displayed as N (%); Continuous variables were digested as median (Q1, Q3) or Mean (SD).

### 3.2 Univariate analyses

In summary, as shown in **Table 2**, several factors, including WC, sex, fatty liver, BMI, HDL, age, HbA1c, alcohol consumption, smoking status, FPG, and VAI were significantly associated with the outcome. Regular exercise and light-to-moderate alcohol consumption did not show a significant association. The risk generally increased with an increase in the values of these variables, except for HDL-c, where the risk decreased (**Table 2**).

**Table 2:** Univariate Cox proportional hazards regression.

	Statistics	HR (95% CI)	P-value
Sex			
Male	8418 (54.48%)	1.0	
Female	7034 (45.52%)	0.40 (0.31, 0.50)	<0.0001
Age, years	43.71 ± 8.90	1.06 (1.04, 1.07)	<0.0001
Fatty liver			
No	12715 (82.29%)	1.0	
Yes	2737 (17.71%)	7.03 (5.71, 8.64)	<0.0001
AST, U/L	17.00(14.00-21.00)	1.01 (1.01, 1.01)	<0.0001
WC, cm	76.47 ± 9.11	1.09 (1.08, 1.10)	<0.0001
BMI, kg/m^2^	22.12 ± 3.13	1.24 (1.22, 1.27)	<0.0001
ALT, U/L	17.00(13.00-23.00)	1.01 (1.01, 1.01)	<0.0001
Regular exerciser			
No	12747 (82.49%)	1.0	
Yes	2705 (17.51%)	0.76 (0.56, 1.02)	0.0654
HbA1c, %	5.17 ± 0.32	54.25 (39.48, 74.56)	<0.0001
TG, mmol/L	0.73(0.50-1.12)	1.88 (1.75, 2.03)	<0.0001
TC, mmol/L	5.13 ± 0.86	1.49 (1.34, 1.66)	<0.0001
HDL-c, mmol/L	1.46 ± 0.40	0.13 (0.09, 0.18)	<0.0001
GGT, U/L	15.00(11.00-22.00)	1.01 (1.01, 1.01)	<0.0001
Smoking status			
Never	9027 (58.42%)	1.0	
Past	2949 (19.08%)	1.66 (1.26, 2.19)	0.0003
Current	3476 (22.50%)	2.59 (2.06, 3.25)	<0.0001
FPG, mmol/L	5.16 ± 0.41	25.52 (18.81, 34.63)	<0.0001
DBP, mmHg	71.58 ± 10.50	1.05 (1.04, 1.06)	<0.0001
SBP, mmHg	114.49 ± 14.97	1.03 (1.03, 1.04)	<0.0001
Alcohol consumption			
None	11801 (76.37%)	1.0	
Light	1754 (11.35%)	0.91 (0.65, 1.26)	0.5608
Moderate	1357 (8.78%)	1.15 (0.82, 1.63)	0.4159
Heavy	540 (3.49%)	2.25 (1.54, 3.28)	<0.0001
VAI	0.78(0.39-1.58)	1.27 (1.24, 1.31)	<0.0001
VAI tertile			
T1	5151 (33.34%)	1.0	
T2	5150 (33.33%)	2.82 (1.85, 4.29)	<0.0001
T3	5151 (33.34%)	7.54 (5.13, 11.07)	<0.0001

**Figure 2:**
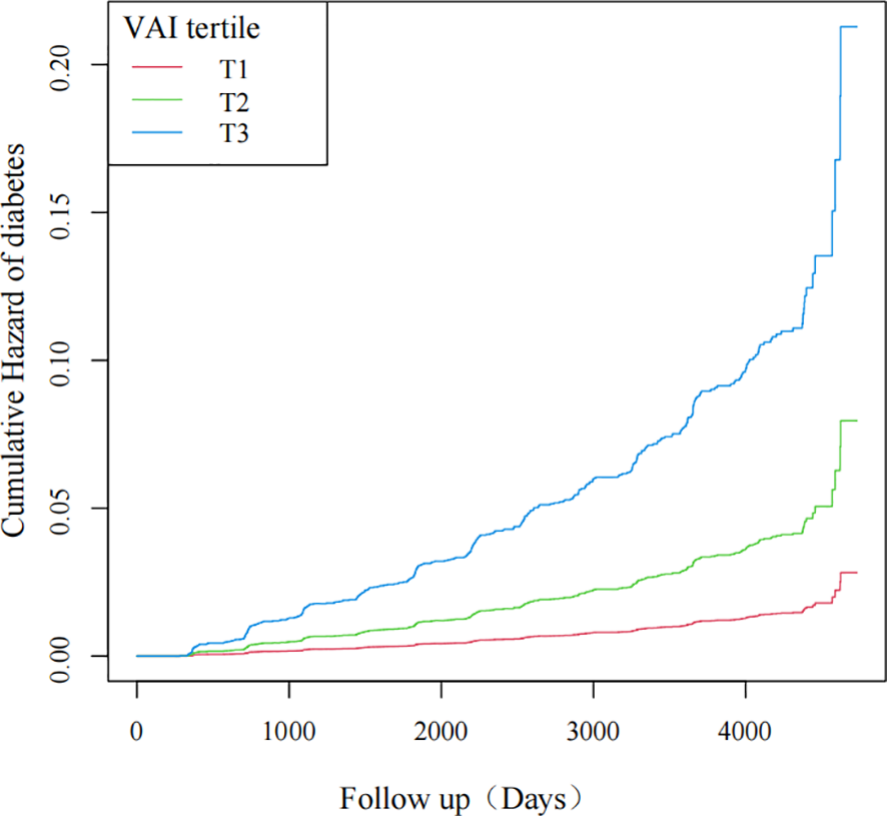
Kaplan-Meier curves for the probability of diabetes.

As the VAI value increased, so did the probability of diabetes, as revealed by the curves. Individuals with the highest VAI values were at the greatest risk of developing diabetes (**Figure 2**).

### 3.3 Multivariate analyses

Because of the collinearity of the DBP variable with other factors, it was excluded from the multivariate Cox proportional hazards regression analysis. The Crude Model illustrated that for each unit increment in the VAI, the risk of diabetes escalated by 27% (HR=1.27, 95% CI 1.24-1.31). In Model I, a similar unit rise in the VAI corresponded to a 24% surge in diabetes risk (HR=1.24, 95% CI 1.20-1.28). Model II indicated a 13% increase in diabetes risk for each unit increment in the VAI (HR=1.13, 95% CI 1.08-1.18). When examining VAI tertiles, the diabetes risk intensified. Specifically, T2 showed a 73% elevated risk (HR=1.73, 95% CI 1.11- 2.72) and T3 showed a 138% heightened risk (HR=2.38, 95% CI 1.46-3.87) in comparison to T1 (**Table 3**).

**Table 3:** Effect of the VAI on diabetes risk in various models.

Exposure	Crude Model (HR, 95% CI, P)	Model I (HR, 95% CI, P)	Model II (HR, 95% CI, P)
VAI	1.27 (1.24, 1.31) <0.0001	1.24 (1.20, 1.28) <0.0001	1.13 (1.08, 1.18) <0.0001
VAI tertile			
T1	1.0	1.0	1.0
T2	2.82 (1.85, 4.29) <0.0001	2.60 (1.67, 4.05) <0.0001	1.73 (1.11, 2.72) 0.0165
T3	7.54 (5.13, 11.07) <0.0001	7.12 (4.51, 11.23) <0.0001	2.38 (1.46, 3.87) 0.0005
P for trend	<0.0001	<0.0001	0.0004

Crude Model: unadjusted.

Model I: adjusted for age and sex.

Model II: adjusted for smoking status, fatty liver, TC, ALT, SBP, age, AST, sex, GGT, regular exerciser, and alcohol consumption.

### 3.4 Sensitivity analyses

To validate our findings, we performed three sensitivity analyses, and all models corroborated the primary results. In Model I, one unit increment in the VAI corresponded to a 14% increase in new-onset diabetes (HR=1.14, 95%CI 1.08-1.20). Model II indicated a 12% increase in new-onset diabetes for each unit rise in the VAI (HR=1.12, 95%CI 1.06-1.19). Finally, Model III demonstrated that each unit rise in the VAI was related to a 13% increase in new-onset diabetes (HR=1.13, 95%CI 1.08-1.19) (**Table 4**).

**Table 4:** Effect of the VAI on diabetes risk in various sensitivity analyses.

Exposure	Model I (HR, 95% CI, P)	Model II (HR, 95% CI, P)	Model III (HR, 95% CI, P)
VAI	1.14 (1.08, 1.20) <0.0001	1.12 (1.06, 1.19) <0.0001	1.13 (1.08, 1.19) <0.0001
VAI tertile			
T1	1.0	1.0	1.0
T2	1.72 (1.05, 2.81) 0.0319	2.13 (1.28, 3.55) 0.0035	1.67 (1.05, 2.64) 0.0287
T3	2.43 (1.42, 4.13) 0.0011	2.71 (1.54, 4.75) 0.0005	2.50 (1.52, 4.13) 0.0003
P for trend	0.0008	0.0012	0.0002

Model I: Non-exercise. Adjusted for AST, smoking status, GGT, SBP, ALT, sex, TC, fatty liver, alcohol consumption, and age.

Model II: Non-alcohol. Adjusted for AST, smoking status, GGT, SBP, ALT, sex, TC, regular exerciser, fatty liver, and age.

Model III: Non-hypertension. Adjusted for AST, sex, GGT, ALT, age, TC, regular exerciser, alcohol consumption, fatty liver, and smoking status.

An E-value was computed to evaluate the impact of unobserved confounding variables. The main outcomes remained solid, except in the presence of an unobserved confounder with an HR exceeding 1.79.

### 3.5 Non-linear analyses

**Figure 3:**
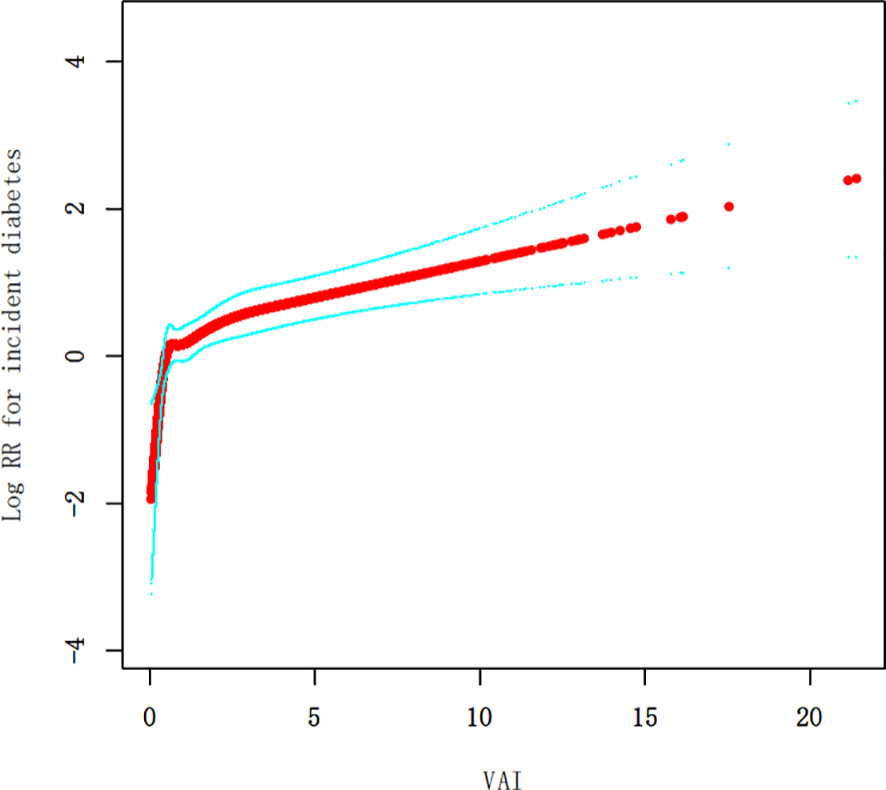
The non-linear link between the VAI and diabetes.

Our research identified a non-linear relationship between the VAI and diabetes (**Figure 3**, **Table 5**). The inflection point of this relationship was observed at a VAI value of 4.67. When the VAI values were less than or equal to 4.67, a unit rise in the VAI corresponded to a 24% surge in new-onset diabetes (HR=1.24, 95% CI 1.12-1.37, p<0.0001). Conversely, for the VAI values exceeding 4.67, new-onset diabetes rose by 5% for a one-unit rise in the VAI, although this correlation was not a statistically significant one (HR=1.05, 95% CI 0.96-1.15, p=0.2893).

**Table 5:** The result of the two-piecewise Cox regression model.

Diabetes incident	HR, 95%CI	P-value
Standard Cox regression	1.13 (1.08, 1.18)	<0.0001
Two-piecewise Cox regression		
Inflection point of the VAI	4.67	
≤4.67	1.24 (1.12, 1.37)	<0.0001
>4.67	1.05 (0.96, 1.15)	0.2893
P for log-likelihood ratio test		0.040

We adjusted fatty liver, SBP, TC, GGT, AST, sex, ALT, age, regular exercise, alcohol consumption, and smoking status.

### 3.6 Subgroup analyses

Analyses of various subgroups revealed no significant interactions between VAI and the onset of diabetes across age, BMI, fatty liver, smoking status, and regular exerciser strata (**Table 6**). The findings indicated that sex could influence the relevance of the VAI to the onset of diabetes. A more robust relation was detected in women (HR=1.40, 95% CI 1.14-1.70, p=0.0010) in comparison to men (HR=1.13, 95% CI 1.08-1.18, p<0.0001).

**Table 6:** Stratified relationship between the VAI and diabetes.

Characteristic	Number of participants	HR (95%CI) P value	P for interaction
Sex			0.0054
Male	8,418	1.13 (1.08, 1.18) <0.0001	
Female	7,034	1.40 (1.14, 1.70) 0.0010	
Age, years			0.6126
18-38	4,842	1.16 (1.05, 1.29) 0.0055	
39-46	5,111	1.10 (1.01, 1.20) 0.0214	
47-79	5,499	1.13 (1.06, 1.20) <0.0001	
Smoking status			0.3239
Never	9,027	1.09 (0.99, 1.20) 0.0768	
Past/Current	6,425	1.15 (1.10, 1.21) <0.0001	
BMI, kg/m^2^			0.2721
<25	12,931	1.15 (1.08, 1.23) <0.0001	
≥25	2,521	1.10 (1.03, 1.18) 0.0027	
Fatty liver			0.9554
No	12,715	1.15 (1.06, 1.24) 0.0010	
Yes	2,737	1.14 (1.08, 1.20) <0.0001	
Regular exerciser			0.7659
No	12,747	1.14 (1.09, 1.20) <0.0001	
Yes	2,705	1.12 (0.99, 1.26) 0.0640	

Note 1: The above stratification was adjusted for smoking status, fatty liver, TC, AST, sex, ALT, GGT, SBP, regular exercise, alcohol consumption, and age.

Note 2: The stratification variable is unadjusted in each instance.

### 3.7 ROC analyses

**Figure 4:**
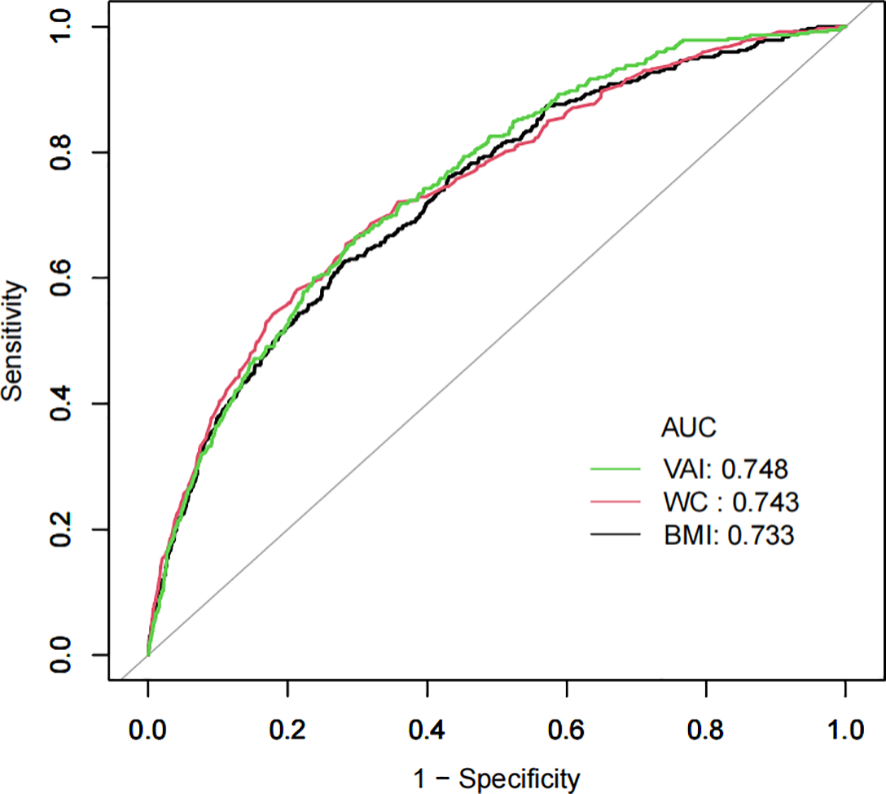
ROC curves for the VAI prediction of diabetes risk.

The potential of the VAI as a predictor of diabetes was evaluated using ROC analysis, as demonstrated in **Table 7** and **Figure 4**, with an AUC of 0.7479 (95% CI: 0.7237-0.7720). Surprisingly, VAI showed the highest AUC compared with BMI and WC, showing a stronger ability to predict diabetes (70.35% specificity, 66.49% sensitivity) (**Table 7**).

**Table 7:** ROC analysis for diabetes prediction.

Variables	AUC	95%CI	Best threshold	Specificity	Sensitivity
BMI, kg/m^2^	0.7327	0.7069-0.7586	23.5285	0.7184	0.6273
WC, cm	0.7425	0.7165-0.7686	81.0500	0.7164	0.6542
VAI	0.7479	0.7237-0.7720	1.3270	0.7035	0.6649

## 4 Discussion

Our comprehensive longitudinal research, conducted in a Japanese cohort, revealed a positive, non-linear link between the VAI and diabetes. This relationship was consistent across all subgroups, with notable sex-based interactions. This is the first study, to the best of our understanding, that identifies a positive correlation between the VAI and the potential for diabetes in normoglycemic Japanese adults.

Studies in the U.S. have shown that the prevalence of diabetes in adults aged 20 years and older is approximately 9.6% (28). Approximately 12% of adults in Japan have diabetes (29). The prevalence of diabetes in our study was 2.41%, which was lower than the prevalence rates reported in previous literature (28, 29), most likely due to the stricter inclusion and exclusion criteria of the original study. The original study excluded patients with fatty liver disease, heavy alcohol intake, any medication use, and prediabetes, all of which are major risk factors for diabetes, resulting in a lower prevalence of the disease (30–33).

Our research corroborated earlier findings of a positive correlation between the VAI and diabetes, a relationship that has been observed in various populations. For instance, Liu conducted a cross-sectional study involving 2,754 Chinese adults aged 20 to 50, and an elevated VAI was shown to be positively connected with diabetes mellitus (20). Alkhalaqi examined a random sample of 1,103 Qatari adult residents aged over 20 years in a cross-sectional study and discovered not only an association between VAI z-scores and the onset of diabetes (OR=1.44; 95% CI 1.24-1.68) but that this relationship was more pronounced in women than in men (21). Koloverou found in a prospective cohort study of 1,049 participants from Greece (ATTICA study) with a 10-year follow-up that a higher VAI greatly increased the chances of developing diabetes by 22% (OR = 1.22; 95% CI: 1.09-1.37), with this association being particularly evident in women (22). Moreover, Zheng conducted a cross-sectional study of 18,745 American adults and observed a non-linear positive trend between the VAI and diabetes, with women showing a stronger risk than men (23). In conclusion, our cohort study not only confirmed the positive VAI and diabetes correlation in a new demographic group, Japanese adults but also revealed a non-linear relationship with a distinct inflection point.

The VAI encompasses both metabolic and physical aspects, potentially serving as an indirect indicator of some unconventional risk factors. These include changes in elevated plasma free fatty acids, enhanced lipolysis, and adipocytokine production, aspects not captured by WC, HDL-c, BMI, and TG when considered individually (34). As such, the VAI could be useful for measuring adipose tissue allocation and features (15, 34), which are related to increased diabetes risk and have an inverse relationship with insulin sensitivity (35).

The exact biological processes linking the VAI and diabetes remain somewhat unclear. One aspect involves the build-up of visceral fat, which is linked to reduced cerebral insulin sensitivity (36). A recently conducted study demonstrated that a 9-month regimen of a high-fiber and low-fat diet with heightened exercise brought about an immediate weight reduction that correlated with increased brain insulin sensitivity (36). Another factor is the inhibitory impact of fatty acid oxidation products on key enzymes involved in glucose breakdown, which means that higher free fatty acid content in plasma, common in insulin resistance and obesity, could worsen impaired glucose metabolism (36). Finally, adipose tissue failing to enlarge normally leads to abnormal fat build-up in visceral areas and even in vital organs such as the muscles, liver, and pancreas, which can disrupt their normal functioning (37).

The results of the subgroup analyses revealed that more associative correlations were observed in women when considering VAI and diabetes risk. Several potential reasons can be identified. First, estrogen, a crucial regulator of metabolic equilibrium and insulin sensitivity, can lessen oxidative stress and immune cell infiltration in adipose tissue and decrease inflammation in white adipose tissue (38, 39). This reduction can mitigate potential ectopic lipids in the liver and skeletal muscle (38, 39). In our investigation, as middle-aged and older women lost estrogen’s protective effects, they became more prone to insulin resistance compared to men. Second, Koutsari proposed that women exhibit a higher prevalence of non-oxidative free fatty acid disposal, which could potentially elevate TG levels, leading to diabetes (40–42). Finally, women, relative to men, have lower fatty acid oxidation and basal lipolysis levels, making them more susceptible to diabetes (43).

Our research suggests that a non-linear correlation exists between the VAI and diabetes, with a VAI value turning point at 4.67. Maintaining a VAI under 4.67 could notably reduce diabetes probability. Yet, once one’s VAI surpasses 4.67, a plateau effect is observed, and merely reducing it below 4.67 does not significantly impact diabetes risk. Thus, it becomes essential to control other risk elements such as smoking and hypertension. The sex-based differences in the relationship indicate a need for a heightened focus on diabetes risk in women. Crucially, managing VAI in women calls for more stringent and proactive measures.

Comparative analyses show that the VAI outperforms common metrics, including BMI and WC. Hameed et al. observed that the VAI had a higher AUC for predicting glycemic control in diabetic patients than BMI and WC, with AUC values of 0.670 for the VAI and 0.491 for BMI (44). Hulkoti et al. (45) found that the VAI had the highest AUC in diabetic microvascular issues (VAI = 0.826, WC = 0.813, BMI = 0.806). Similarly, our results strengthen these findings and clarify the unique contribution of the VAI to diabetes risk including comparisons with other adiposity measures such as BMI and WC. Its ability to integrate anthropometric and metabolic parameters provides a more nuanced understanding of an individual’s health status, making it a valuable tool in clinical practice for identifying those at risk for diabetes.

### 4.1 Study strengths and limitations

There are some merits to this study. First, it elucidated the connection between VAI and diabetes in normoglycemic adults in Japan. This investigation utilized a cohort study, enabling a clearer understanding of the cause-and-effect relationship between the VAI and diabetes. Second, this research treated the VAI as both a categorical and a continuous variable to reveal a non-linear association and a saturation effect. Finally, this research, being a national population-based cohort study with an extended follow-up duration, boasts broad coverage and excellent sample representativeness.

Certain limitations of this study warrant mention. First, the research is a secondary analysis of already available data and therefore, it does not include metrics related to diet and family history. Second, failure to include glucose tolerance and random blood glucose testing in the determination of diabetes may have caused the diabetes prevalence to be under-evaluated. Finally, the research demographic was comprised of Japanese adults, making the results less applicable to other ethnicities. Given the low incidence of diabetes, future studies should consider longer follow-up periods or larger sample sizes to ensure adequate numbers of events for the subgroup analyses.

## 5 Conclusion

Our cohort research, which involved 15,452 participants from NAGALA between 2004 and 2015, confirmed the non-linear and positive link between the VAI and diabetes in normoglycemic Japanese adults. The significantly more relevant results for women versus men suggest an increased need to concentrate on diabetes risk in women. A significant reduction in diabetes risk could potentially be achieved by keeping one’s VAI below 4.67.

## Data Availability

The original contributions presented in the study are included in the article/supplementary material. Further inquiries can be directed to the corresponding authors.
